# Fetal Hepatic Response to Bovine Viral Diarrhea Virus Infection in Utero

**DOI:** 10.3390/pathogens7020054

**Published:** 2018-06-06

**Authors:** Susan E. Morarie-Kane, Natalia P. Smirnova, Thomas R. Hansen, Jessica Mediger, Lyle Braun, Christopher Chase

**Affiliations:** 1 Department of Veterinary and Biomedical Sciences, South Dakota State University, Brookings, SD 57007, USA; suek@ncesd.org (S.E.M.-K.); jessica.mediger@sdstate.edu (J.M.); braun_lyle@elanco.com (L.B.); 2 Animal Reproduction and Biotechnology Laboratory, Department of Biomedical Sciences, College of Veterinary Medicine and Biomedical Sciences, Colorado State University, Fort Collins, CO 80523, USA; Natalia.Smirnova@sidelinesoft.com (N.P.S.); Thomas.Hansen@ColoState.edu (T.R.H.)

**Keywords:** bovine viral diarrhea virus, persistent infection, liver, hepatic tolerance, Kupffer cells

## Abstract

Non-cytopathic bovine viral diarrhea virus (ncp BVDV) can cause persistent infection (PI) in animals infected in utero during early gestation. PI animals shed the virus for life and are the major source of the virus in herds. The mechanism responsible for BVDV immune tolerance in the PI fetus is unknown. We assessed the impact of BVDV infection on the fetal liver. Dams were inoculated with ncp BVDV at gestational day 75. Fetal liver samples were collected at necropsy, 7 and 14 days post-maternal-BVDV inoculation. BVDV antigen was not detected in the liver at gestational day 82 (7 days post-maternal inoculation). However, at 14 days post-maternal inoculation, BVDV was detected by immunohistochemistry in fetal Kupffer cells. Flow cytometry analysis showed a higher percentage of hepatic immune cells expressed MHC I and MHC II in BVDV-infected fetal liver (as compared to uninfected controls). Immunofluorescence was used to identify Kupffer cells, which were positive for BVDV antigen, near populations of CD3+ lymphocytes. The identification of BVDV in the fetal liver Kupffer cells at 14 days post inoculation is interesting in the context of establishment of tolerance in persistent infection. These data indicate the presence of a hepatic immune response to fetal infection.

## 1. Introduction

Bovine viral diarrhea virus (BVDV) causes wide spread infection of ruminants and leads to significant economic losses for cattlemen on a global scale [1,2]. BVDV causes clinical disease with pathology ranging from subclinical symptoms to respiratory disease, gastrointestinal illness, reproductive infections, immune suppression, mucosal disease, and/or death [3]. The severity of illness and the physiological systems affected are strain dependent [4]. BVDV virus that infects the dam during pregnancy crosses the placenta and infects the developing fetus with a time-dependent outcome. Early in the first trimester, the infection can result in embryo-fetal loss, and in the third trimester, the infection results in specific fetal immune responses [3,4,5]. However, if a pregnant dam is infected with non-cytopathic BVDV (ncp BVDV) between days 40–120 of gestation, the fetus may become persistently infected (PI) [6,7]. This time frame is critical for the establishment of the fetal persistent infection, because at this stage of gestation the fetal immune system is not mature enough to mount the appropriate immune response needed to clear the virus, and the calf becomes tolerant to the virus. This tolerance is strain specific [8], and the BVDV PI calf sheds the virus for the rest of its life. While the phenomenon of persistent infection, and the specific window of opportunity in gestation are well documented, the mechanism of BVDV tolerance in PI fetuses is still unknown. 

The in utero route of fetal infection has been the focus of a number of research studies [9,10,11,12]. BVDV virus in maternal blood transverses the placenta (placentome), into the fetal umbilical vein, and to the fetal liver [9,10,11,12]. Subsequently, blood is routed to the right fetal atrium of the heart. Therein, BVDV has direct access to fetal liver cells prior to entering the fetal heart. Notably, BVDV antigen was previously observed at 14 days post-maternal infection in the fetal liver [9], but the cell type was not conclusively identified. Identification of the cell type, and further characterization of the innate immune response within the fetal liver could provide important insight into BVDV pathology and persistent infection. 

The fetal liver in mammals, including the calf, is the site of the ontogeny of the immune system. Fetal liver hematopoietic stem cells move from the yolk sac to the liver early in development at ~40 days [13,14]. In the fetal calf, the length of time that the liver is a major hematopoiesis, and lymphopoiesis, organ is not well defined, but in humans, the liver remains an important immune organ throughout gestation and postnatally [15]. Fetal hematopoietic stem cell production in the fetal liver peaks in bovine fetuses at 12–24 weeks of gestation [16]. 

Resident liver immune cells, specifically Kupffer cells, are an important part of the innate immune response [17,18]. Kupffer cells, found in the liver sinusoids, are the first population of macrophages to encounter antigens such as bacterial debris, endotoxins, and viruses taken in with digestive content [19]. Kupffer cells are paramount arbitrators of the immune response in the hepatic environment. They exhibit both excitatory and tolerant measures in their interactions with leukocytes and are capable of secreting inflammatory mediators including interleukin (IL)-1, IL-6, and tumor necrosis factor (TNF)-α [20], as well as tolerogenic cytokines such as IL-10 [21]. Tolerance is the prevailing response of the liver so that the immune system does not react inappropriately to digestive content antigens [22]. Early studies showed that intraportal application of antigens induced antigen-specific systemic tolerance [23,24], suggesting that the immune response established in the liver may have an extended influence on the immune response in other tissues. 

Hepatic immune tolerance is intriguing with respect to BVDV persistent infection because if cells within the fetal liver are infected with maternally transmitted virus, these cells could present viral antigen to lymphocytes during critical times in development. An in vivo study to evaluate various parameters in fetal and maternal pathology in ncp BVDV infection provided a rare opportunity to investigate these liver-tolerance mechanisms [25]. Pregnant heifers were inoculated with ncp BVDV at 75 days in gestation to establish parameters which would yield persistently infected fetuses. Several papers have subsequently described results from this study including: the maternal innate immune response to infection [25], the pathology of the fetal central nervous system [26], and the pathology of trabecular modeling in fetal long-bone [27]. To begin to characterize the interaction of BVDV with the fetal liver, fresh and fixed liver samples were taken at the necropsy of fetuses collected by cesarean section at specific time-points in gestation after maternal infection with BVDV. Samples were analyzed by flow cytometry, immunohistochemistry, and immunofluorescence to identify infected fetal cells, as well as immunological presentation proteins MHC I and MHC II, and the presence of CD3 positive lymphocytes in the liver. Clarification of the innate immune response and identification of BVDV-infected cells within the fetal liver could provide additional insight into the mechanism of BVDV persistent infection.

## 2. Results

### 2.1. Detection of Bovine Viral Diarrhea Virus

**Figure 1:**
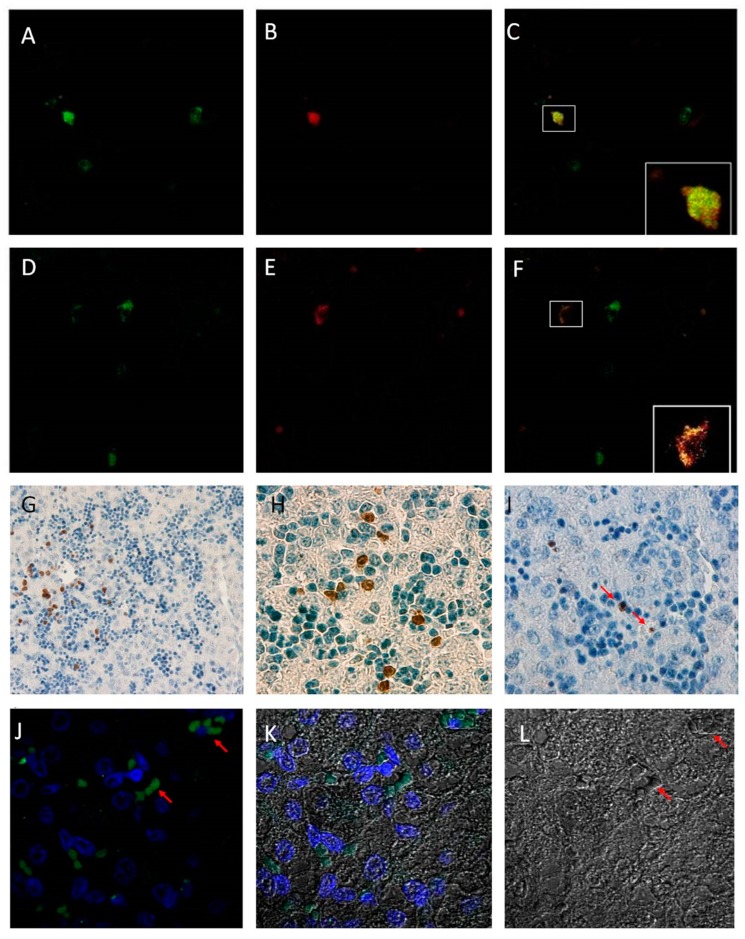
Panel (**A**–**F**) Representative Confocal images co-localize Mac387 positive cells (green) with BVDV 15C5 positive cells (red). Merged images display a double positive cell in each field. Co-localization is magnified in insets. Images were taken at 40× magnification; (**G**–**I**) immunohistochemistry of Mac387 and BVDV in persistently infected fetal livers at gestational day 89, 14 days post maternal-infection; (**G**,**H**) are representative images of Mac387 positive cells in the sinusoid of fetal liver tissue. 200× and 800× magnification respectively; (**I**) is a representative image of BVDV positive cells (indicated with red arrows) stained with 15C5 antibody at 400× magnification. (**J**–**K**) are representative immunofluorescence of CD3. Paraffin embedded section of bovine liver at gestational day 89. Antigen retrieval: Heat 10 min in citrate buffer pH 6. Anti-CD3 marker labeled with Goat-Anti-Mouse conjugated FITC. DAPI staining for nuclei. All confocal images at 100× magnification; (**J**) fluorescent labels only; (**K**) fluorescent imaged layered over Disk Confocal imaging section (DICS); (**L**) DICS only. Red arrows are representative corresponding CD3 positive lymphocytes.

In fetuses collected at 82 days in gestation, seven days post-maternal BVDV infection, BVDV antigen was not detectable by immunohistochemistry (IHC) in fixed fetal liver tissue. However, BVDV was detected at 14 days post-maternal-infection (day 89 of gestation) in the liver. BVDV-infected cells were located primarily in liver sinusoids and near central veins (Figure 1I). BVDV positive cells were also located in close proximity to numerous hematopoietic precursor populations, which were quite numerous at this stage in fetal development. Hepatocytes and hematopoietic precursors were not infected with BVDV as determined by IHC. Additionally, BVDV was readily detected by IHC in all subsequent infected fetal liver samples collected at 96, 190 and 245 days in gestation (data not shown). 

### 2.2. Kupffer Cell Characterization

Isolated hepatic immune cells were cultured for several days to evaluate the purity of the cell culture and to observe the cell morphology and phagocytic activity. All cultures displayed cell morphology that was consistent with macrophage-like cells and were capable of phagocytosis when assessed by 1 h incubation with fluorescent microspheres (data not shown).

### 2.3. Immunohistochemistry and Immunofluorescence

Kupffer cells were identified in paraffin-embedded fixed fetal hepatic tissue samples by both immunohistochemistry and immunofluorescence with antibody Mac387, which is specific to the L1 marker found on Kupffer cells, macrophages, and circulating monocytes. Kupffer cells are distinguishable from infiltrating monocytes based on their histomorphology and location. The cells identified as Kupffer cells were L1+ (as identified by antibody Mac387) and distributed primarily in the sinusoidal regions of the fetal liver (Figure 1G,H). Kupffer cells were observed in all fetal liver tissue samples (both control and PI tissues). 

Immunofluorescent staining for BVDV antigen with 15C5 monoclonal antibody was also used to co-localize the viral antigen with Kupffer cells in paraffin-embedded sections in PI tissue samples (Figure 1A–C). Control tissue samples were positive for the L1 marker, but did not display the co-localization of the viral antigen (images not shown). Confocal microscopy revealed a co-localization of both antibody markers in select cells in PI tissue samples. Notably, Kupffer cells in PI tissue samples were not uniformly positive for BVDV antigen at 14 days post-maternal infection, as some Kupffer cells were only positive for the L1 marker (Figure 1D–F); however, no other hepatic cell population was identified as being positive for BVDV antigen.

Staining of the liver sections with CD3 antibody revealed presence of CD3 positive lymphocytes by immunofluorescence in paraffin-embedded tissues clustered in numerous, small aggregate populations near Kupffer cells at 89 days in gestation in both PI and control samples (Figure 1J). 

### 2.4. MHC I/MHC II Expression

**Figure 2:**
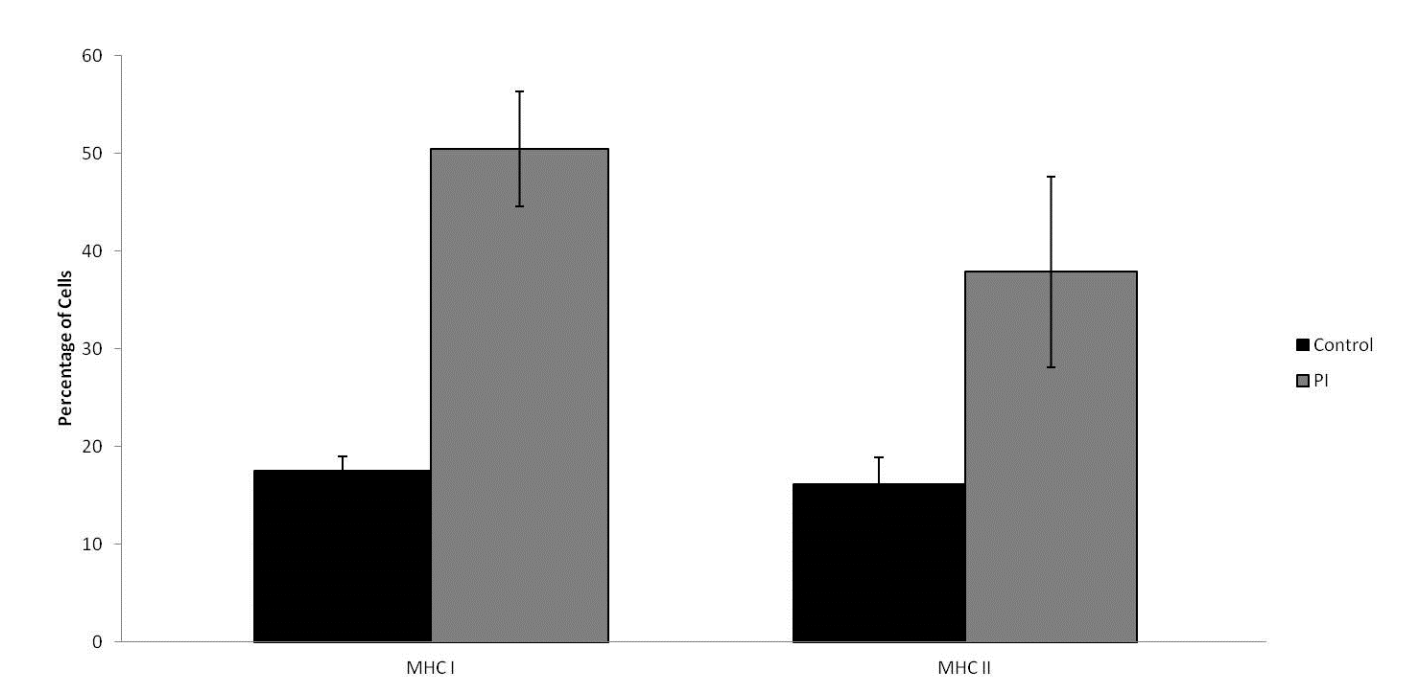
Flow cytometry of primary cells isolated from fetal liver samples at 14 days post-maternal-infection with BVDV. There was a statistically significant increase in the percentage of cells expressing MHC I and MHC II. Error bars shown are representative of one standard deviation.

Primary hepatic immune cells were isolated and stained for antigen presentation molecules, MHC I and MHC II. Flow cytometry analysis of the stained cells revealed that more than twice as many cells (*p* = 0.00041) were expressing MHC I in persistently infected cell samples. Comparatively, 17.54% of cells were expressing MHC I in control animal samples to 50.47% in PI cell samples (Figure 2). The percentage of cell expressing MHC II on the cell surface was also nearly doubled (*p* = 0.01743), in cells from BVDV positive fetal livers; MHC II was expressed on 16.14% of cells in control samples, while 37.86% of cells from infected fetal liver samples expressed MHC II (Figure 2).

## 3. Discussion

The importance of the fetal liver to the development of BVDV persistent infection in vivo has received minimal attention; however, this unique series of experiments with fetal liver tissues provided an opportunity to investigate interaction of hematopoiesis in the liver with the flavivirus, BVDV. At 14 days post-maternal infection, Kupffer cells, present in the fetal liver, were BVDV positive and in close proximity to CD3+ T-cells. Fetal liver also had an increase in the number of antigen presenting (MHC II) cells. 

Currently, there is not a consensus around the mechanism for the fetal immune dysfunction that results in BVDV persistent infection. It is known that PI animals are born when a pregnant dam becomes infected with ncp BVDV virus between gestational days 40 and 120, the actual mechanism of BVDV-established tolerance is not fully understood [28]. An understanding of the mechanism of immune-dysfunction, which results in viral persistence, would be of considerable value. A targeted intervention (i.e., treatment or vaccination) could have a significant economic impact. BVDV is one of the most costly diseases of cattle that leads to abortion, infertility, immune suppression, respiratory disease, and mucosal disease. Most animals become exposed through contact with other acutely infected or PI animals in the herd that are shedding the virus. 

The route of entry for any pathogen can have dramatic implications on the pathogenesis and immune response in the host. The physiological organization of maternal–fetal circulation provides a unique route for the BVDV to enter the developing fetus. BVDV is able to infect cells across the placentomal barrier between the mother and fetus, which allows viral entry into the fetal blood stream [10,11,12,13,14]. Once inside the fetal circulation, the umbilical vein transports the blood supply through the fetal liver carrying nutrients and oxygen for the fetus [13]. BVDV, along with oxygen and other nutrients, enters the fetal circulation and can be detected in fetal tissues by IHC as early as 14 days post-maternal infection as shown in this study. Confocal microscopy, used to identify Kupffer cells within the fetal liver, at 89 days in gestation, detected cells positive for BVDV, as well as the L1 marker identifying Kupffer cells [20]. Infection of Kupffer cells with the virus may contribute to the development of persistent infection.

Kupffer cells are capable of presenting antigen to lymphocytes, and BVDV infection does not compromise antigen presentation ex vivo [29]. However, in the microenvironment of the liver, the presentation often results in tolerance rather than activation [22,30,31]. Risadle and co-authors evaluated the hepatic response to a transient infection with BVDV and reported an increase in the number of Kupffer cells in tissues collected from infected animals [20]. The present study confirms a sinusoidal distribution of fetal Kupffer cells during development, as demonstrated in histopathology sections stained with the same monoclonal MAC387 antibody for detection of Kupffer cells as the one used in the published study [20]. Kupffer cells express MHC I and MHC II on their cell surface. This study found an increased number of MHC I, and MHC II, expressing cells in PI fetal liver as compared to uninfected controls. While other cell types may be likely to express MHC I, MHC II is somewhat more exclusive in it distribution, and its increase is indicative of an increase of the number of professional antigen presenting cells in PI fetal liver. An increase in the number of Kupffer cells could not be conclusively determined by this analysis; however, the data provided here, as well as the data reporting an increase in the number of Kupffer cells in transient infection with BVDV [20], support a pathological increase in the number of Kupffer cells in infected liver tissues.

An increase in the number of antigen presenting cells, like Kupffer cells, would only account for one side of a tolerance mechanism. Antigen-presenting cells present antigens to lymphocytes. Lymphocytes have been identified in circulation early in development [32]. In a developing bovine fetus, the liver is a center for hematopoiesis and lymphogenesis [13]. Here, CD3+ lymphocytes were identified in the 89-day fetal liver in both persistently infected and control animals. The presence of lymphocytes in the liver is an important component of the liver tolerance model because the liver, like the lymph node, is involved in primary activation of naive T cells [32,33]. Notably, the location where a lymphocyte is educated has tremendous implications on the outcome of its response to antigen presentation because lymphocytes activated in the lymph node differentiate in distinctly different ways than those activated in the liver. Importantly, activation in hepatic tissues has been shown to provide a strong bias towards immune tolerance [34]. However, while CD3+ lymphocytes were present in proximity to fetal Kupffer cells, it is not clear if the CD3+ lymphocytes observed in this study were mature enough to respond to antigen or persist as memory cells.

The tolerogenic nature of the fetal hepatic microenvironment is an intriguing consideration for the establishment of BVDV persistent infection during gestation. Kupffer cells produce tolerogenic cytokines such as IL-10 and TGF-β that promote immune suppression profiles in lymphocytes [21,35,36]. CD8+ T cells specifically, are activated when they are presented an antigen in the context of MHC I, by an antigen presenting cell. The activation results in the production of cytokines such as IFNγ and TNFα and initiation of cytolytic mechanisms [37]. However, CD8+ T cells are greater in number in the liver than anywhere else in the body, and antigen presentation to CD8+ lymphocytes, in the liver, results in tolerance rather than cytotoxicity [36,38].

Kupffer cells express PD-L1, a ligand for CD8+ T cell receptor, on their surface. PD-L1 is upregulated when type I interferon production decreases [30,39]. PD-1 ligands (PD-Ls) on antigen-presenting cells have been shown to switch off autoreactive T cells and induce peripheral tolerance [40]. Kupffer cells, presenting BVDV antigen and PD-L1, might extinguish any active immune recognition and therein evade an initial immune response. 

Inflammation changes conditions in the hepatic microenvironment and can stimulate immune activation. Inflammatory cytokines like IFN-α serve as a danger signal in the liver and can incite immune responses such as the generation of acute phase proteins [41]. During pregnancy, it is critical that inflammatory signals remain low to protect the developing fetus from the maternal immune system and vice versa. However, the pathogenesis of persistent infection in neonate calves may also merit further research into the hepatic microenvironment. 

The data presented here is only ground work for further research; however, it is plausible that the introduction of BVDV to the fetus in the liver may have a prolonged impact on the nature of the immune response to the virus. The interaction of infected Kupffer cells with lymphocytes was observed in this study, but identification of the specific lymphocyte subset represented, and the specific cytokine signals present will require future study.

## 4. Methods

### 4.1. Animals and Sample Collection

This study was a part of a larger study that was performed at Colorado State University (CSU) and approved by the CSU IACUC Committee [24]. The larger study was comprised of forty-six (46) heifers that were synchronized and artificially inseminated. The animals were then randomized and 23 of the 46 were infected with a type 2 ncp BVDV strain (96B2222) at 75 days in gestation to generate PI fetuses and the remaining 23 animals were controls [24]. Each heifer in the PI group received intranasal inoculation of a 2 mL aliquot of the virus stock at 4.4 log_10_ TCID_50_/mL as described previously [24] or a sham inoculation with 2 mL of culture media (control group). 

Fetuses were collected by cesarean section, from randomly assigned heifers at days 82, 89, 96, 190 and 245 days in gestation (7, 14, 21, 115 and 170 days post-maternal infection respectively) as previously described [24]. Liver samples were collected from the fetuses in the day 82 and 89 groups (*n* = 4 control and 4 PI fetuses for each collection day) to assess hepatic response to fetal infection with BVDV. Fresh liver samples were split for primary culture and flow cytometry analysis or fixed for immunohistochemistry and immunofluorescence analysis.

### 4.2. Kupffer Cell Isolation

To isolate the Kupffer cells from fetal livers, a sample of fresh fetal liver (approximately 1 cm^3^) was manually homogenized in PBS and 0.5% heparin (10 units/mL) followed by incubation with trypsin and collagenase for 10 min in filtered stomacher bags (Thermo Fisher, Waltham, MA, USA). The fluid fraction containing cells was removed from the opposite side of the filter and remaining tissues were flushed twice with PBS containing heparin to capture any residual cells. All fluid fractions containing cells were pooled and treated with a low isotonic lysing solution for 1 min to lyse red blood cells. Osmolarity was restored with a 10× Hanks balanced salt solution. The cell suspension was centrifuged to reduce volume and cells were resuspended in sterile PBS. The cell suspension was layered on 65% Percoll gradient to separate immune cells. Isolated hepatic immune cells were rinsed several times with PBS, passed through a 0.45 um cell strainer, and counted for viability. A 96-well plate was seeded with 5 × 10^5^ cells/well in 100 µL of buffer (PBS with 1% Bovine Serum Albumin) for flow cytometry, and the remaining cells were resuspended in 6-well plates at 5 × 10^5^ cells/mL in RPMI 1640 (Invitrogen/Thermo Fisher, Waltham, MA, USA) with 15% BVDV-Free Fetal Bovine Serum (Atlanta Biologicals, GA, USA) and penicillin, streptomycin and gentamicin (Sigma, St. Louis, MO, USA) and cultured at 5% CO_2_/37 °C to observe cell morphology and assess phagocytic activity. 

### 4.3. Phagocytic Activity Evaluation

To evaluate phagocytic activity, cells were incubated in 6-well plates for one hour to allow the majority of the cells to adhere to the tissue culture plate. After which, the media was drawn off, and the adherent cells were incubated with small fluorescent spheres suspended in RPMI1640 without serum. Phagocytosis was confirmed by fixing cells to the tissue culture plate with 2% paraformaldehyde instead of detaching and using UV microscopy where the number of beads ingested by Kupffer cells was counted. 

### 4.4. Flow Cytometry

To evaluate immune markers, the cells that had been seeded in 96 well round-bottom plates were centrifuged, supernatant was discarded, and cells were resuspended in PBS with 5% Bovine Serum Albumin, which also served as blocking solution for flow cytometry. The cells were then incubated for 30 min with primary anti-bovine antibodies (diluted 1:200) for anti-bovine MHC I (VMRD, USA) or MHC II (VMRD), washed with PBS three times, incubated for 30 min with a FITC-conjugated goat anti-mouse secondary antibody (AbCam, Cambridge, MA, USA) (diluted 1:200), fixed with 2% paraformaldehyde, and then analyzed by flow cytometry. Control samples from each fetus were incubated in blocking solution without primary antibodies. Data were collected for 10,000 events on a Cyan-ADP Flow cytometer (Dako, Ft. Collins, CO, USA) and analyzed with Summit software (Dako, Ft. Collins, CO, USA).

### 4.5. Immunohistochemistry

Fresh tissue was fixed in 10% formalin for 24–48 h followed by 70% ethanol for 48 h before paraffin wax embedding. Five-micron (5 µm) sections were cut and mounted to lysine-charged slides. For staining procedure sections were deparaffinized in three changes of Citrisolv (Fisher, USA), and rehydrated in an incremental series of 100% isopropyl alcohol, 100% ethyl alcohol, 90% ethyl alcohol, 70% ethyl alcohol, and finally deionized water. Rehydrated tissue was rinsed with PBS and processed for antigen retrieval with heat for 20 min in 100 mM Citrate buffer at pH 6. After incubation with the primary antibody for either Kupffer cell or BVDV antigen, an avidin-biotin commercial kit (Vector Laboratories, Burlingame, CA, USA) was used with a DAB peroxidase stain (Vector Laboratories, USA). Antibodies 15C5 for BVDV Erns (IDEX, Westbrook, ME, USA) and MAC 387 (AbCam, USA) for Kupffer cells were diluted in blocking buffer 1:200. Table 1 lists the antibodies and the corresponding dilutions used. Control slides were processed in parallel without primary antibody.

### 4.6. Immunofluorescence 

Fixed tissues were processed, mounted, and deparaffinized and the same protocol described above for immunohistochemistry was used for antigen retrieval in citrate buffers. Sections were then blocked for 1 h in PBS with 5% goat serum and incubated for 1 h at room temperature with the same primary antibodies used for immunohistochemistry that were specific for BVDV antigen, and Kupffer cells. Mouse anti-bovine CD3 antibody MM1A (VMRD) was used to stain lymphocytes. Antibodies were diluted in blocking buffer (see table for dilutions). After that sections were rinsed in blocking buffer, followed by incubation with fluorescent-conjugated secondary antibodies also diluted in blocking solution (1:200) for 1 h. Slides were rinsed, covered with cover slips, and read by Confocal microscopy.

### 4.7. Statistical Analysis

Mean CVs were computed from flow histograms for each of the four control and four PI samples analyzed by flow cytometry for both the MHC I and MHC II expression. Data were analyzed by a Student’s *t*-test to determine significance of the difference in tissue expression of MHC I and MHC II in control and PI liver samples. Differences between groups were considered significant if probability values of *p* < 0.05 were obtained.

## Figures and Tables

**Table 1:** Antigens and Antibodies Used for Cell Analysis by Flow Cytometry (FC), Immunohistochemistry (IHC), or Immunofluorescence (IFA).

Target	Cells Identified	Antibody Clone	Source	Isotype	Application	Dilution	Reference
MHC I	PBMC	H58A	VMRD	IgG2a	FC	(1:200)	Davis 1987 [42]
MHC II	APC, PBMC	H42A	VMRD	IgG2a	FC	(1:200)	Davis 1987 [42]
L-1	Macrophage, Monocyte, Kupffer Cells	MAC387	AbCam	IgG1	IHC	(1:200)	Cope 1990 [43]; Risalde 2011 [20]
CD3	T cell Lymphocytes	MM1A	VMRD	IgG1	IFA	(1:200)	MacHugh 1998 [44]
BVDV	BVDV Infected Cells (viral antigen)	15C5	IDEX	IgG2	IHC, IFA	(1:200)	Bazler 1995 [45]; Cornish 2005 [46]; Montgomery 2008 [47]
